# Pulmonary Hemodynamic Changes with Nitric Oxide or Oxygen in a Patient with Asplenia, Single Right Ventricle, and Total Anomalous Pulmonary Venous Connection after Fontan Procedure

**DOI:** 10.1155/2018/3736254

**Published:** 2018-11-25

**Authors:** Hideharu Oka, Kouichi Nakau, Aya Kajihama, Masaya Sugimoto, Hiroshi Azuma

**Affiliations:** Department of Pediatrics, Asahikawa Medical University, Asahikawa 078-8510, Japan

## Abstract

Asplenia syndrome is frequently complicated by a total anomalous pulmonary venous connection. Pulmonary venous obstruction, following total anomalous pulmonary venous connection surgery, is one of the risk factors for morbidity and mortality. In some patients, the pulmonary vasculature is abnormal even in the absence of clinical evidence of pulmonary venous obstruction. We hypothesized that a change in the pulmonary hemodynamics could indicate the abnormality of pulmonary vein in a patient with asplenia, single right ventricle, and total anomalous pulmonary venous connection, following Fontan procedure. Here, we present a case of asplenia, single right ventricle, total anomalous pulmonary venous connection, and right pulmonary venous obstruction in which evidence of a potential left pulmonary venous obstruction was obtained following the administration of inhaled nitric oxide and oxygen.

## 1. Introduction

Asplenia syndrome is frequently complicated by total anomalous pulmonary venous connection (TAPVC). Pulmonary venous obstruction (PVO), following TAPVC surgery, is one of the risk factors for morbidity and mortality. The indexed pulmonary vein sum is smaller in patients with asplenia syndrome than in those without. Furthermore, patients with asplenia syndrome have a thin-walled pulmonary vein [1–3]. The atrial wall is thick, and a direct anastomosis between the atrial wall and the thin-walled pulmonary venous confluence is not particularly desirable [4]. The pathology of PVO after TAPVC repair is indicated by vessels exhibiting fibrous intimal layer hyperplasia associated with some medial layer hypertrophy [5]. In some patients, the intimal hyperplasia involves the pulmonary vein ostia without anastomotic stenosis [6]. Furthermore, in patients with single ventricle and TAPVC, the pulmonary vasculature is abnormal, even in the absence of clinical evidence of PVO [7]. Hence, in a patient with asplenia, single ventricle, and TAPVC, the abnormality of pulmonary vein might occurs easily. Following Fontan procedure, the abnormality of pulmonary vein may have an influence over a patient's hemodynamic parameters. However, it has previously been difficult to diagnose the abnormality of pulmonary vein during a patient's lifetime. We hypothesized that a change in the pulmonary hemodynamics could indicate the abnormality of pulmonary vein. Here, we present a case of asplenia, single right ventricle, TAPVC, and right lower PVO after Fontan procedure, in which evidence of the abnormality of the left pulmonary vein was demonstrated based on the measurements of hemodynamic parameters and responsiveness to inhaled nitric oxide (NO) and oxygen.

## 2. Case Presentation

**Figure 1:**
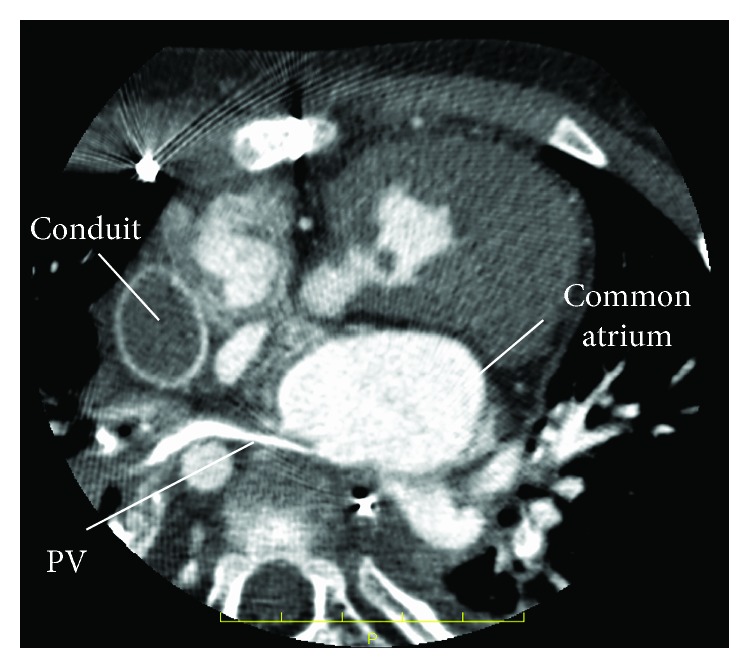
The right lower pulmonary venous obstruction occurred because of the compression of vertebra. PV: pulmonary vein.

**Figure 2:**
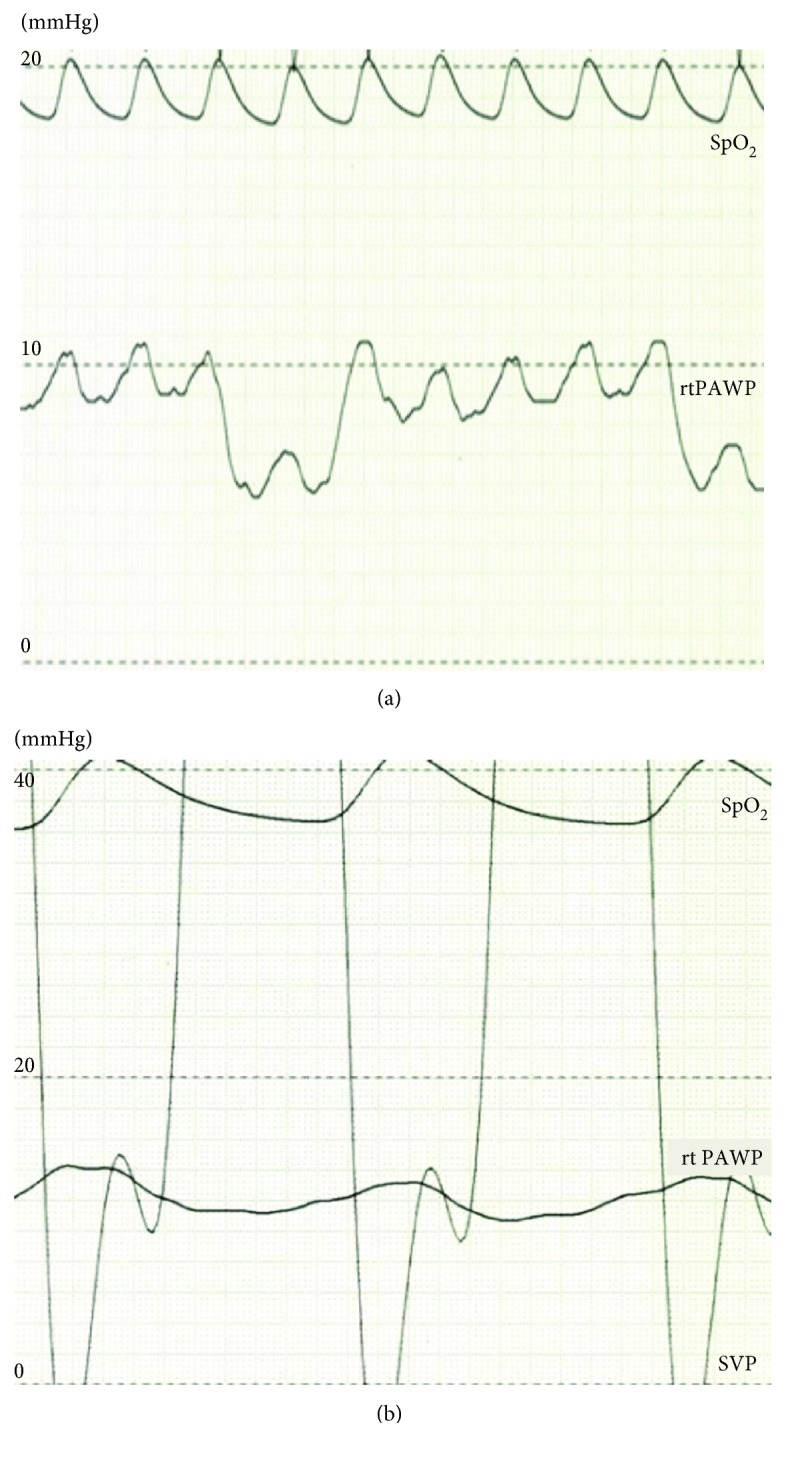
Pulmonary wedge pressure and SVEDP waveform. The pulmonary wedge pressure before NO test in (a) and after NO test in (b). SpO_2_: oxygen saturation; PAWP: right pulmonary arterial wedge pressure; SVP: single ventricle pressure.

**Figure 3:**
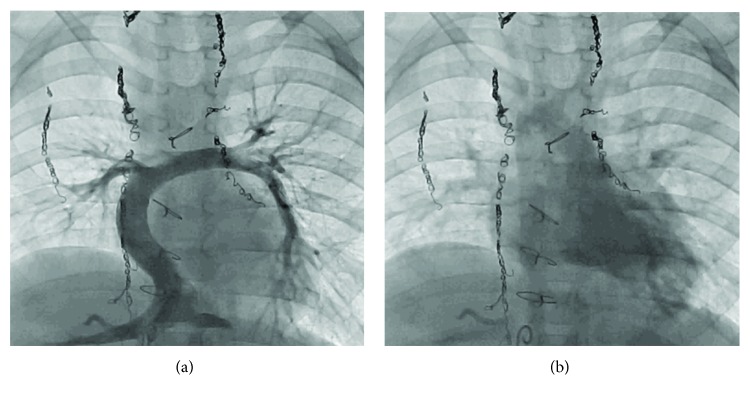
Inferior vena caval angiography. The pulmonary artery is shown in (a), and there is no delay in excretion of the contrast agent in (b).

**Figure 4:**
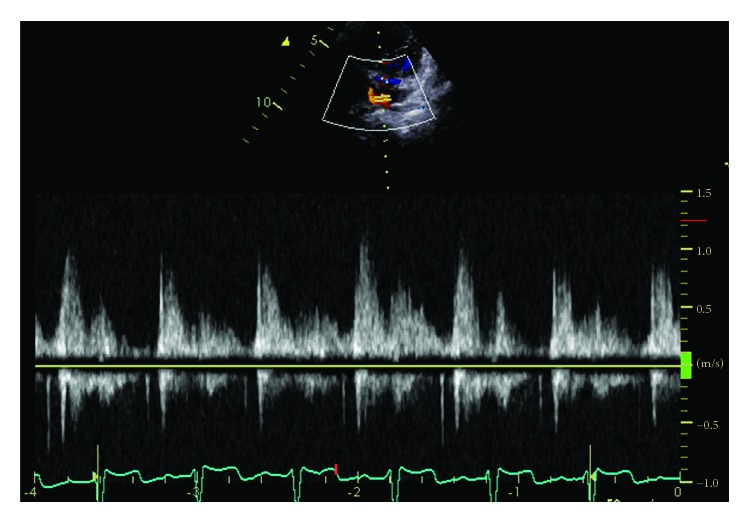
There was no acceleration of blood flow, and stenosis was not indicated at the surgical anastomosis between the pulmonary venous confluence and the atrium in pulmonary vasodilator testing.

A 4-year-old girl was previously diagnosed with asplenia, single right ventricle, and supracardiac TAPVC. She underwent pulmonary artery banding at the age of 1 month, followed by a bidirectional Glenn procedure and TAPVC repair at the age of 5 months. She did not demonstrate symptoms of PVO, and there was no evidence of delay in the excretion of contrast media in cardiac catheter examination before Fontan procedure. Extracardiac Fontan palliation was subsequently performed at the age of 19 months. At the age of 3 years, although right lower PVO occurred because of the compression of the vertebra, she did not demonstrate symptoms of PVO (Figure 1). At the age of 4 years, she underwent a cardiac catheterization to evaluate her hemodynamic parameters after Fontan procedure (Table 1). She was taking oral aspirin, warfarin, and enalapril. After a routine hemodynamic assessment, NO and oxygen tests were performed. A pigtail catheter was placed in the single ventricle, and a wedge catheter was positioned in the pulmonary artery. We performed the pulmonary vasodilator examination in the same way as when doing the examination in patients with pulmonary hypertension [8]. Subsequently, 20 ppm NO was administered via face mask. After 5 minutes of NO administration, her hemodynamic parameters were measured. Following a washout period of 5 minutes, 100% oxygen was administered, and after 5 minutes of oxygen administration, her hemodynamic parameters were again measured. To measure the pulmonary arterial wedge pressure and pulmonary arterial pressure accurately, the waveform was monitored carefully (Figures 2(a) and 2(b)). After the administration of NO, the single-ventricle end-diastolic pressure, bilateral mean pulmonary arterial pressure, and cardiac index did not change. Oxygen saturation did not change at 95%, and pulmonary resistance index decreased from 2.1 to 1.0 Um^2^. However, the right lower pulmonary arterial wedge pressure increased from 8 to 12 mmHg, and the left lower pulmonary arterial wedge pressure increased from 7 to 9 mmHg. The cardiac index remained almost the same. Similarly, following the administration of oxygen, the single-ventricle end-diastolic pressure did not change; however, the right lower pulmonary arterial wedge pressure increased from 8 to 14 mmHg and the left lower pulmonary arterial wedge pressure increased from 7 to 12 mmHg. The bilateral mean pulmonary arterial pressure increased from 13 to 15 mmHg. Although angiography was performed after the inhalation of NO and oxygen, there was no evidence of left pulmonary venous obstruction or delay in the excretion of contrast media (Figures 3(a) and 3(b)). A systemic-to-pulmonary shunt was also nonexistent. On echocardiography, there was no acceleration of blood flow at the left pulmonary vein or surgical anastomosis between the pulmonary venous confluence and the atrium. There was also no stenosis of the atrioventricular valve. Despite the inhalation of NO and oxygen, there was no acceleration of blood flow, and stenosis was not identified at the surgical anastomosis between the pulmonary venous confluence and the atrium (Figure 4).

## 3. Discussion

We administrated inhaled NO and oxygen in a patient with asplenia, single right ventricle, TAPVC, and right lower PVO after Fontan procedure and noted the changes in her hemodynamic parameters. The reduction of pulmonary vascular resistance is useful for hemodynamics in a patient with Fontan procedure. But it is unknown whether the effect of NO or oxygen is valid or not in a patient with Fontan procedure having pulmonary venous abnormality. If the single-ventricle end-diastolic pressure increases, the cause of increasing pulmonary arterial wedge pressure is considered to be single-ventricle dysfunction. However, because single-ventricle end-diastolic pressure did not increase in the present case, the increasing pulmonary arterial wedge pressure and pulmonary arterial pressure were attributed to either a stenosis at the surgical anastomosis or a PVO. Our patient did not exhibit signs of stenosis at the surgical anastomosis between the pulmonary venous confluence and the atrium on echocardiography. Although there was evidence of right lower pulmonary venous obstruction, our patient did not recognize the left lower PVO. Even if pulmonary blood flow was limited owing to the right lower pulmonary venous obstruction, there was no change in the pulmonary arterial pressure as the blood passed through other normal pulmonary veins. The pulmonary arterial wedge pressure also does not increase because the pulmonary blood flow is accommodated by other pulmonary blood vessels, as long as the other vessels are devoid of any abnormalities. Hence, the pathology of increasing pulmonary arterial wedge pressure and mean pulmonary arterial pressure in our patient was postulated to be associated with the abnormality of the left pulmonary vein. We supposed that the postcapillary levels of the pulmonary veins would have been obstructed in this case because the stenosis at the surgical anastomosis and accelerated blood flow of pulmonary veins were not detected. The condition of PVO after TAPVC repair results from the development of fibrous hyperplasia of the intimal layer of the vessel, associated with some hypertrophy of the medial layer [5]. Pulmonary vasculature is abnormal even in the absence of clinical evidence of PVO in patients with single ventricle and TAPVC [7]. Hence, in a patient with asplenia, single ventricle, and TAPVC, the abnormality of pulmonary vein may occur easily. In such patients, other pulmonary blood vessels may not be able to compensate for the increase in pulmonary blood flow by an expansion of the pulmonary artery at the level of the precapillary venules. This leads to an increase in the pulmonary arterial wedge pressure and mean pulmonary arterial pressure and is an important condition in patients undergoing Fontan procedure. Central venous pressure is an important hemodynamic parameter in patients undergoing this procedure, and it depends on the pulmonary arterial pressure. Dilatation of the pulmonary artery is one approach to establish good Fontan circulation. In fact, many studies have discussed the effectiveness of pulmonary vasodilators in the establishment of good Fontan circulation. Aggressive use of combination pulmonary vasodilator therapy is occasionally recommended to achieve good Fontan circulation [9]. However, in patients with the abnormality of pulmonary vein, expanding the pulmonary artery may promote pulmonary congestion and therefore may not allow the establishment of good Fontan circulation. We would like to examine these types of patients who have an abnormality of the pulmonary vein in the future. Our opinion is that a patient with asplenia and TAPVC after Fontan procedure should be considered carefully.

In conclusion, we found evidence of an abnormality of the pulmonary vein following the administration of inhaled NO and oxygen in an asymptomatic patient with asplenia, single right ventricle, TAPVC, and right lower PVO after Fontan procedure. Our findings suggest that in patients with asplenia and TAPVC, there may be a possibility of developing PVO.

## Figures and Tables

**Table 1:** Hemodynamic data in baseline.

SVC pressure (mmHg)	15	SVEF (%)	67
IVC pressure (mmHg)	15	%SVEDV (%*N*)	75
Conduit pressure (mmHg)	15	RsI (Um^2^)	23.6
SVP (mmHg)	105/e9	RpI (Um^2^)	2.1
rt PAP (mmHg)	13	Cardiac index (L·min^−1^·m^−2^)	2.42
lt PAP (mmHg)	13		
rt lower PAWP (mmHg)	8		
lt lower PAWP (mmHg)	7		
AOP (mmHg)	101/58 (72)		

e: end-diastolic pressure; SVC: superior vena cava; IVC: inferior vena cava; SVP: single ventricle pressure; rt PAP: right pulmonary arterial pressure; lt PAP: left pulmonary arterial pressure; rt PAWP: right pulmonary arterial wedge pressure; lt PAWP: left pulmonary arterial wedge pressure; AOP: aortic pressure; SVEF: single ventricle ejection fraction; SVEDV: single-ventricle end-diastolic volume; %*N*: percent of normal; RsI: systemic resistance index; RpI: pulmonary resistance index.
